# Multimodal boiling dataset with synchronized acoustic, optical, and thermal measurements under steady-state and transient heat loads

**DOI:** 10.1016/j.dib.2024.110582

**Published:** 2024-06-11

**Authors:** Hari Pandey, Changgen Li, Han Hu

**Affiliations:** Department of Mechanical Engineering, University of Arkansas, Fayetteville, AR 72701, USA

**Keywords:** Pool boiling, Critical heat flux, Acoustic emissions, High-speed imaging

## Abstract

Boiling is a high-performance heat dissipation process that is central to electronics cooling and power generation. However, there exists a practical limit of boiling heat transfer known as the critical heat flux (CHF), beyond which significant performance degradation is observed. Understanding the physical mechanism that triggers CHF is essential to meet the increasing cooling demands driven by power densification and device miniaturization. However, the high dimensionality, stochasticity, and dynamicity of the boiling process have led to strong challenges in the experimental characterization and modeling of boiling CHF. As such, high-frame rate, high-resolution, multi-physics boiling datasets are critical to advancing the fundamental understanding of boiling heat transfer. To this end, this paper presents a multimodal boiling dataset consisting of synchronized thermal, acoustic, and optical signals collected from five different heater surfaces under two distinct heat load conditions. With its high sampling frequency, diverse signal types, large data volume, and detailed recorded information, this dataset provides valuable "data blood" for the field of thermal crisis monitoring. This dataset will not only promote fundamental research on bubble dynamics during boiling but also support the implementation of advanced monitoring technologies in industrial applications such as power electronics, motors, data centers, and power plants.

Specifications Table

**Table:** 

Subject	Mechanical Engineering
Specific subject area	Phase change phenomena for thermal control have been a key interest in many engineering fields. Boiling, a two-phase heat transfer, is the specific research area.
Type of data	Phantom Cine Files (.Cine) boiling videos which contain 1000 representative images for each power load for steady-state cases. Text file (.txt) extracted from raw file for acoustic emission (AE) sensor. LabVIEW measurement files (.lvm) for both boiling acoustics by using hydrophones, and temperature data obtained from thermocouples reading were acquired. MATLAB file for temperature data analysis for calculating heat flux
Data collection	The datasets were collected from different nanostructured surfaces subjected to pool boiling experiments. These surfaces include polished copper, polished microchannel, and copper foams electrodeposited at different pHs, which are, pH 0, pH 10, and pH 12. These pool boiling tests were run in two different heat loading conditions including steady state and transient ramp-up heat load. A multimodal sensing dataset was collected where temperature, heat flux, and acoustics were collected in a synchronized manner using LabVIEW, boiling videos via Phantom Camera Control (PCC), and AE data using AEwin software developed by Physical Acoustics Corporation.
Data source location	• Institution: University of Arkansas • City/Town/Region: Fayetteville, AR • Country: United States Latitude and longitude for collected data: 36.0687º N, 94.1748º W
Data accessibility	Repository name: Pool Boiling Dataset: Synchronized Acoustic, Optical, and Thermal Measurements for Steady-State and Transient Heat Loads. Data identification number: Direct URL to data: https://dataverse.harvard.edu/dataset.xhtml?persistentId=doi:10.7910/DVN/6GLGC6
Related research article	H. Pandey, H. Mehrabi, A. Williams, C. Mira-Hernández, R. H. Coridan, and H. Hu, “Acoustic sensing for investigating critical heat flux enhancement during pool boiling on electrodeposited copper foams,” Appl. Therm. Eng., vol. 236, p. 121807, Jan. 2024, doi: 10.1016/j.applthermaleng.2023.121807.[1]

## 1 Value of the Data

• The dataset provides first-of-kind multimodal boiling data from 0 to CHF conditions which contain thermal (temperature, heat flux), liquid acoustics (pressure waves recorded by hydrophones), transient elastic waves through solids (acoustic emission), and boiling image data (depicting bubble dynamics, bubble behavior, bubbling frequency, etc.) during steady state and transient boiling for different boiling surfaces including plain copper, hierarchical copper foams and microchannels.
• The data contains detailed labels, which can serve as a benchmark for data scientists to develop machine learning algorithms and evaluate them. Additionally, it can aid thermal engineers in developing effective monitoring systems to prevent TMS from sudden faults or crises while employing high-performance cooling.
• The boiling video data contains 1000 representative images for each heat flux during steady-state conditions that can be used in the classification of boiling stages into early nucleate boiling regime, stages dominated by vapor columns and formation of mushroom bubbles, and transition boiling after critical heat flux (CHF) triggering. The differences in bubble dynamics in each heat flux will facilitate an in-depth study of the role of boiling surfaces and provide surface optimization.
• The data provides acoustic features from hydrophone signals and acoustic emission (AE) waves provided with a label of each heat flux during steady-state and transient boiling which can be used to correlate the acoustics-based thermal monitoring of the phase change cooling systems.

## 2 Background

Boiling is an ultra-efficient heat dissipation process critical to the thermal management of power electronics, data center racks, nucleate reactors, and jet engines, among others. Nevertheless, boiling heat transfer is limited by a catastrophic point of failure known as the critical heat flux (CHF). Beyond CHF, a continuous vapor layer is developed to blanket the heater surface, leading to significantly reduced heat transfer coefficient and rapid heat accumulation. Real-time monitoring of the boiling process is thus critical to ensuring safe operations. Meanwhile, the improvement of CHF will rely on the breakthrough in the fundamental understanding of the physical mechanism that triggers CHF. Multiphysics, high-resolution, high-frame rate boiling datasets are required for both advancing fundamental understanding of boiling and developing real-time monitoring and fault detection technologies.

In the early days, boiling was explored solely based on thermal data using temperature sensors [2]. With the development of visualization techniques, different optical imaging techniques have been explored to understand boiling dynamics including endoscopes [3], high-speed cameras [4], and infrared measurements [5], etc. The combination of visual and thermal data provides direct validation for theoretical models and correlations related to bubble dynamics, bubble behavior, and boiling heat transfer mechanisms. However, the addition of new physical phenomena like acoustics supports distinguishing the different boiling regimes on varying heat fluxes [6] and detecting the approach of critical heat flux [7]. Several acoustic sensors have been used in boiling characterization which provides leverage on non-intrusive monitoring, including acoustic emission (AE) sensors [[8], [9], [10], [11], [12]], hydrophones [7,[13], [14], [15], [16], [17], [18]], and microphones [6,19,20]. The multimodal data of optical, acoustic, and thermal signals provides a comprehensive understanding of complex boiling phenomena which has been used to probe transport mechanisms during CHF [1], heat flux predictions [[21], [22], [23]], and boiling regime detections [24].

In the present study, a multimodal dataset is generated during pool boiling experiments under both steady-state and transient heat load conditions. During the tests, the provided dataset includes all the regions of two-phase heat transfer which includes the initial free convection, early bubble nucleation, rapid bubble generation, and vapor blanketing. The availability of different boiling images extracted from the provided videos denotes the different boiling stages which are convection boiling, nucleate boiling, CHF state, transition, and film boiling. The dataset acquired during these tests can be used for real-time smart temperature control and monitoring of the thermal systems. The multimodal data including images, temperature, heat flux, acoustic emission (AE), and hydrophone acoustics, can provide data-based preventive measures while developing a real-time thermal monitoring system. The developed system based on actual thermal scenarios will put a top-end value on the factor of safety and can save more energy which otherwise will be limited due to steady-state-based thermal solutions. The CHF data is synchronized with two different types of boiling acoustics that can bolster effective CHF prediction. Finally, the data helps in developing non-intrusive fault detection in the timely monitoring of the two-phase cooling systems by correlating acoustic signatures with thermal data.

## 3 Data Description

**Fig. 1:**
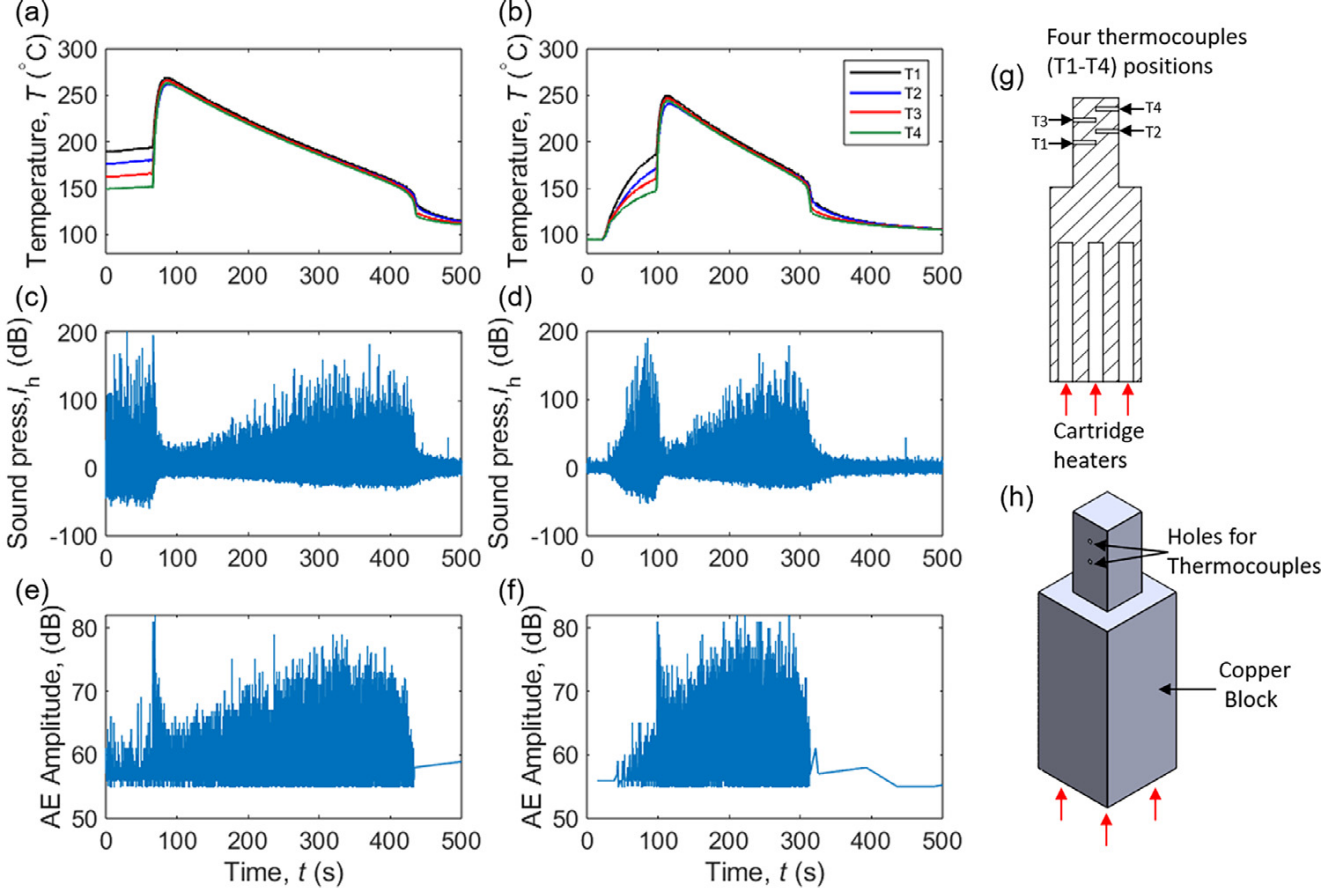
Synchronized temperature and acoustic signals during the CHF condition from a representative steady-state test (a, c, e) and a representative transient test (b, d, f) on a pH 10 electrodeposited copper foam surface. (a-b) Temperature histories in the copper block with T1, T2, T3, and T4 showing thermocouple recordings; (c-d) Sound pressure measured using hydrophones submerged in the pool; (e-f) AE amplitude measured using the AE sensor attached to the bottom of the boiling chamber; (g) the location of thermocouples with a sectional view of copper block; and (h) isometric view of copper block depicting holes for thermocouples on one side.

The data repository [25] comprises various data file types which are in .txt format for “AE”, .lvm for “Hydrophone”, .cine for “Videos”, .lvm for “Temperature” and a Heat_flux.m MATLAB code file for calculating heat flux for each temperature data file. Each of these different data files has a source name starting with “Tr” for Transient and “SS” for SteadyState power load application. A full data file looks like “Power load type_File type_Surface Structure_Power load_File format. Here, “Power load type” is “Tr” for Transient and “SS” for SteadyState. “File type” contains “AE_sensor”, “Images” in the form of pool boiling videos, “Temperature” and “Hydrophone”. For “Surface Structure”, the surface types are “pH-0″, “pH-10″, “pH-12”, “Polished Cu”, and “Polished MC” respectively. Because the “Transient” dataset represents a ramp-up of 0 to CHF boiling state, it only has a single file as power load “CHF”. This can be observed in Fig 1(b) where the transient temperature dataset collected from four thermocouples starts near the liquid saturation point up to the CHF and returns to its original position again as power is turned OFF after CHF initiation. But for the “SteadyState”, the data file for “Power load” varies from 8 to 16 data points depending upon the type of surface. A representative power load data during CHF is plotted for the steady-state case in Fig. 1(a) where the thermocouple reading starts in the range of 150–190 °C and spikes during CHF which will return to water saturation temperature after power is turned OFF (Table 1).

**Table 1:** Summary of test conditions over boiling surfaces with a representative file name.

No	Boiling Surface	Hydrophone		AE sensor		Thermocouple		Optical Videos	
		Transient	Steady	Transient	Steady	Transient	Steady	Transient	Steady
1	Polished Cu	Tr_Hydrophone_Polished_Copper_CHF.lvm	SS_Hydrophone_Polished_Copper_15.lvm	Tr_AE_Sensor_Polished_Copper_CHF.txt	SS_AE_Sensor_Polished_Copper_CHF.txt	Tr_Temperature_Polished_Copper_CHF.lvm	SS_Temperature_Polished_Copper_15.lvm	NA	SS_Images_Polished_Copper_15.cine
2	Polished MC	Tr_Hydrophone_Polished_ MC_CHF.lvm	SS_Hydrophone_Polished_MC_CHF.lvm	Tr_AE_Sensor_Polished_ MC_CHF.txt	SS_AE_Sensor_Polished_MC_CHF.txt	Tr_Temperature_Polished _MC_CHF.lvm	SS_Temperature_Polished_MC_CHF.lvm	NA	SS_Images_Polished_MC_CHF.cine
3	PH 0	Tr_Hydrophone_PH0_CHF.lvm	SS_Hydrophone_PH0_30.lvm	Tr_AE_Sensor_PH0_CHF.txt	SS_AE_Sensor_PH0_CHF.txt	Tr_Temperature_PH0_CHF.lvm	SS_Temperature_PH0_30.lvm	NA	SS_Images_PH0_30.cine
4	PH 10	Tr_Hydrophone_PH10_CHF.lvm	SS_Hydrophone_PH10_15.lvm	Tr_AE_Sensor_PH10_CHF.txt	SS_AE_Sensor_PH10_CHF.txt	Tr_Temperature_PH10_15.lvm	SS_Temperature_PH10_CHF.lvm	NA	SS_Images_PH10_15.cine
5	PH 12	Tr_Hydrophone_PH12_CHF.lvm	SS_Hydrophone_PH12_CHF.lvm	Tr_AE_Sensor_PH12_CHF.txt	SS_AE_Sensor_PH12_CHF.txt	Tr_Temperature_PH12_CHF.lvm	SS_Temperature_PH12_CHF.lvm	NA	SS_Images_PH12_CHF.cine

Note*:* Transient represent transient boiling. Steady represent steady state boiling.

Here Fig. 1a, b shows four temperature readings T1, T2, T3, and T4 from thermocouples placed inside the heating block which is in the direction of the boiling surface starting close to cartridge heaters. That is, T1 is close to the heater, T4 is close to the boiling surface and others are in that order as shown in section figure and isometric view in Fig. 1g, h. Fig. 1c, d represents the sound pressure recorded by the hydrophone. These are the pressure waves recorded inside the liquid pool during the boiling process. Hydrophones are optimized for a specific range of frequencies depending on the application. Boiling acoustics are due to pressure waves generated in the formation of vapor bubbles, during rapid bubble growth, and bubble collapse [26]. Fig. 1e, f shows the AE amplitude measured by the AE sensor during the boiling process. Acoustic emissions are the transient mechanical vibrations that are produced within a body when it experiences sudden external forces. AE sensors detect the stress waves within the material due to changes in its internal structure. In boiling, the AE sensor responds to the acoustics propagated through the solid walls of the chamber. AE signatures have different properties which are differentiated on various parameters based on their nature, location, severity, time, rate, etc. These AE parameters are AE amplitude, AE energy, AE duration, AE rise time, AE count, AE hits, and so on.

**Fig. 2:**
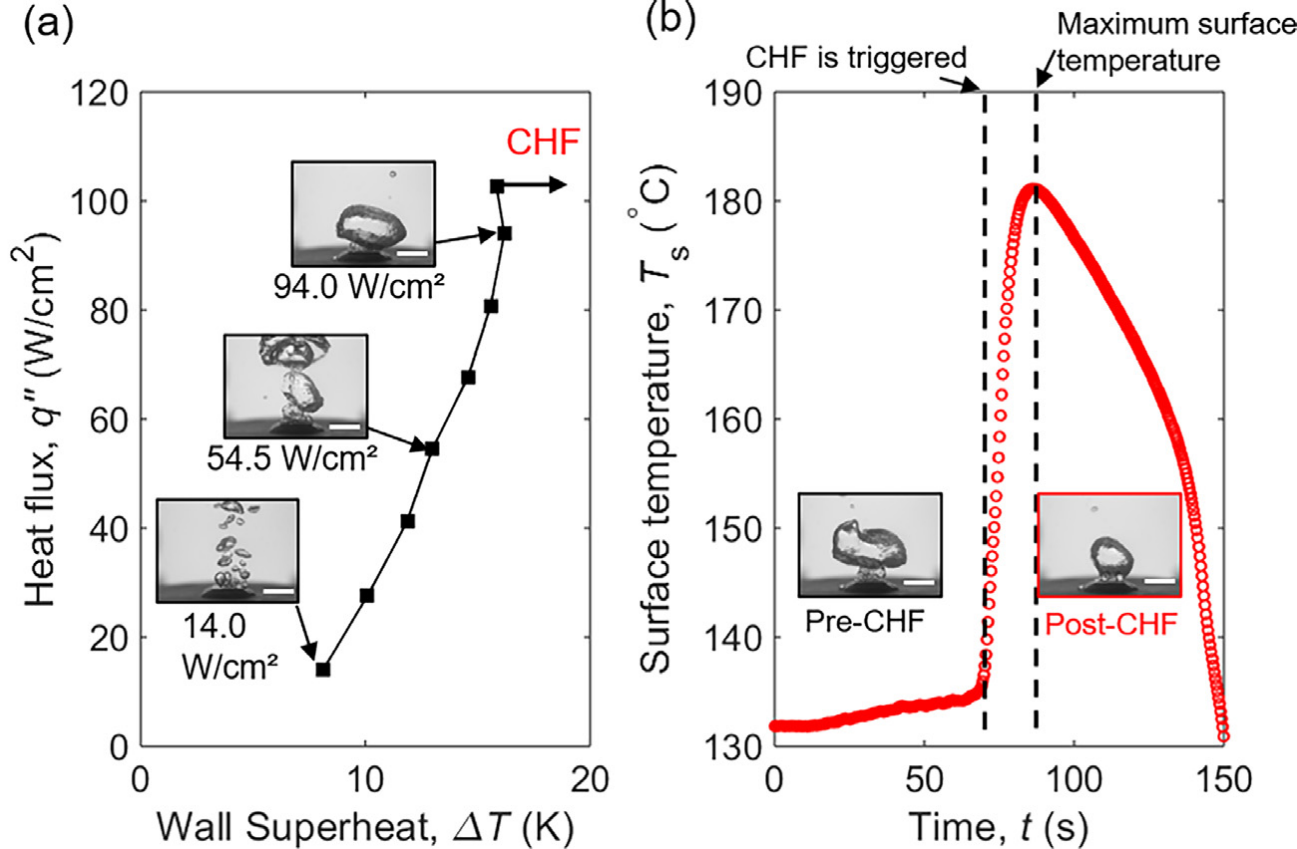
(a) The steady-state boiling curve of water on a polished copper surface with representative optical images at different heat fluxes. (b) Temperature spike observed during the CHF condition. The scale bar represents 10 mm.

Fig. 2 shows the steady-state boiling curve which has 8 power load data points for a polished copper surface with representative optical images including early bubble nucleation, rapid bubble generation and active bubble merging, and formation of mushroom clouds. For others, there are 14,12,11, and 16 heat load application points for pH 0, pH 10, pH 12, and Polished MC respectively. Fig. 2b shows the surface temperature extrapolated from the recorded temperatures (T1, T2, T3, and T4) inside the heater section as shown in Fig. 1a, b. As CHF is triggered, the power supply is turned off in under 3 s to prevent the system from thermal runaway. Due to vapor blanketing, the surface temperature reaches a maximum point and starts to plummet as there is no steady power applied to the system. The inset on Fig. 2b shows the pre-CHF and post-CHF boiling images showing the differences between bubbles generated into mushroom clouds and a stubby region of film attached to the surface respectively.

The actual size of each applied power boiling video dataset can be up to 32 GB which requires a lot of space. As such, no boiling video for transient cases is uploaded to the data repository. These data can be provided on request. The videos for “Steady State” have representative 1000 frames recorded at 3000 frames per second(fps) with an image resolution of 832 × 600. Overall, the complete dataset includes individual files for acoustic sound signals obtained from hydrophone in .lvm format, AEdata extracted from MISTRAS DAQ in .txt format, temperature data in .lvm format for thermocouples present in copper block heater, the liquid pool and vapor in the boiling chamber, and videos in .cine format recorded from a high-speed camera. The heat flux data can be extracted using Heat_flux.m MATLAB file with a corresponding temperature.lvm data file. Here, an example of a pH-0 copper foam sample as a boiling surface for steady-state heat loading is explained below for the actual dataset obtained from sensors including hydrophones, AE sensors, high-speed camera, and thermocouples. The transient data also have similar data measurement parameters as steady state one but the difference is transient data has a single data file which contains 0 to CHF situation and video data are not included in it.

• SS_AE_Sensor_PH0_15.TXT

The data contains the transient acoustic waves recorded by the AE sensor with the testing date, time, and data acquisition in seconds format. They are extracted for the source raw file (not included here) which has a different file type as .DAT. The different AE parameters such as AE RISE, AE COUNT, AE ENERGY, AE DURATION, and AE AMPLITUDE values are recorded as time progresses.

• SS_Hydrophone_PH0_15.LVM

The data contains the sound pressure values obtained from the hydrophones placed at different locations. The sampling rate, date, and time values are available in the file header. Here, Sound Pressure and Sound Pressure_0 represent the reading from two hydrophones.

• SS_Temperature_PH0_15.LVM

The data contains the temperature reading by six different thermocouples where Temperature_0, Temperature_1, Temperature_2, Temperature_3, and Temperature_4 are reading from four t-type thermocouples placed inside the copper block heater. Based on the temperature reading, the highest reading is close to the cartridge heaters whereas the lowest temperature reading is for the thermocouple closest to the boiling surface. The rest of the two, that is, Temperature_4 and Temperature_5 are the temperature readings for vapor inside the boiling tank and liquid pool respectively. Here the liquid pool is near saturated state fluctuation around 99–100 °C.

• SS_Images_PH0_15.CINE

The data contains the high-speed boiling videos for 1000 frames in sequential order which can be extracted to boiling images in .tif or .jpeg format using CineViewer (CV) software. Here, only the 1000 representative boiling images are provided for a specific type of boiling surface (pH-0) at a heat flux level of 15 W/cm².The steady-state pool boiling images are recorded at 3000 fps with an image resolution size of 832 × 600 due to limited camera RAM and storage limitations.

## 4 Experimental Design, Materials, and Methods

### 4.1 Experiment setup

**Fig. 3:**
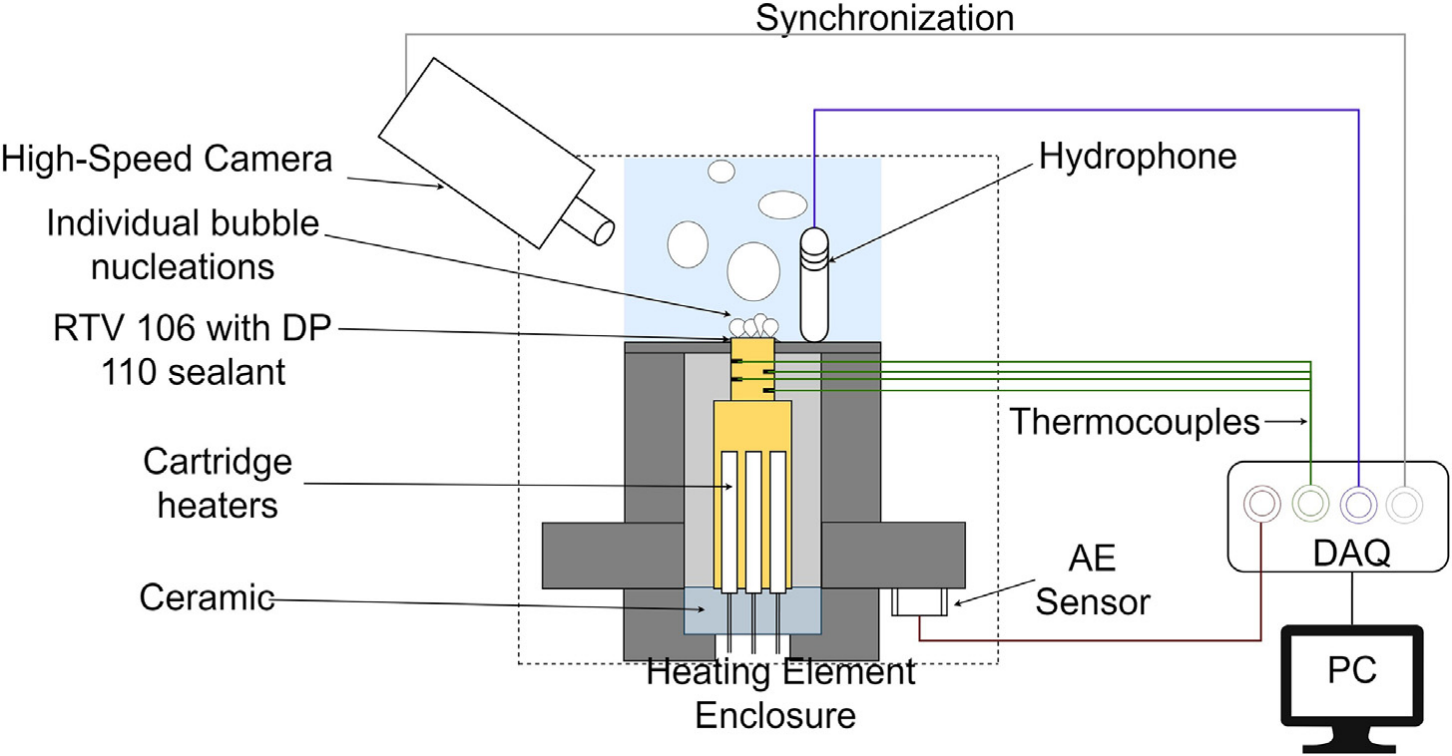
Schematic drawing for the heating element enclosure of pool boiling test facility illustrating the different multimodal sensing consisting of high-speed imaging, sound signals from hydrophones, acoustic emissions from AE sensor, and thermal data from thermocouples.

The pool boiling test facility has a closed boiling tank with a condensing loop, a heating enclosure, and a multimodal sensing system. The boiling tank is made up of polycarbonate side walls and ceiling, and a PEEK base which houses the heating enclosure. The inner volume of the boiling chamber is 6.7 × 6.7 × 7 inch^3^ which is filled with deionized water to approximately 4 inches from the chamber floor. There are two condensing loops a. graham condenser (Ace glass 5953–106) open to atmosphere b. copper coiled condenser inside the boiling chamber. Graham condenser is used during degassing the chamber for the first 30 min where a coiled condenser is used in pressure control during the boiling tests. Fig. 3 represents the section of complete pool boiling with heating element enclosure and multimodal sensing of acoustics, heaters, temperatures, and high-speed imaging. The heating element consists of a copper block with a 1 cm x 1 cm surface area with 9 insertion holes from the bottom for cartridge heaters (Omega Engineering HDC19102). The cartridge heaters are powered using a DC power supply (Magna-Power SL200–7.5). An in-house built PEEK enclosure houses the copper block which sits on a ceramic base and is properly insulated using fiberglass. The junction between the PEEK enclosure and copper block is sealed using RTV 106 with DP 110 sealant. The temperature data are measured using four T-type thermocouples from Omega Engineering TJ36-CPSS-032U-6 evenly spaced at the copper block heater for extrapolating the surface temperature and heat flux calculation. Two immersion heaters are provided in the boiling chamber (not seen in the figure below) for degassing and keeping the liquid pool saturated. The boiling videos are collected using a Phantom VEO 710 L highspeed camera. An LED light source from Advanced Illuminated was used for backlighting in visualizing the bubble behaviors during different stages of boiling. Boiling sounds traveled through liquid media are obtained from submerged HTI-96-Min hydrophone from High Tech, Inc. with a built-in preamplifier. A MISTRAS R3a AE sensor with a resonant response at 29 kHz is attached to the bottom of the boiling tank for recording the transient stress waves propagated due to boiling acoustics. A NI DAQ system with chassis cDAQ-9178 is used with several modules including temperature module NI 9210 for collecting temperature data, sound and vibration module NI 9230 for collecting the hydrophone data, and voltage input module NI 9239 for pressure monitoring during the pool boiling tests. A MISTRAS USB AE Node developed by Physical Acoustics Corporation is used to collect the AE signals from the R3a AE sensor.

### 4.2 Data acquisition

**Fig. 4:**
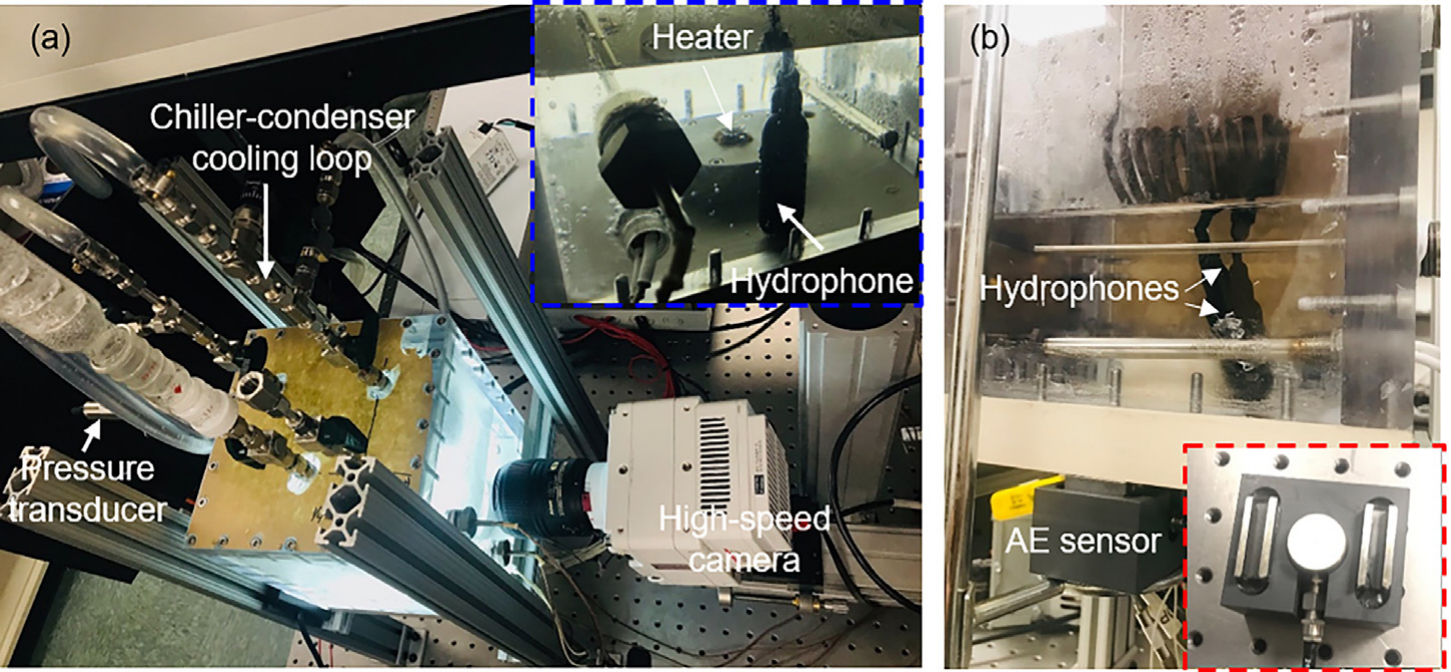
Pool boiling test facility with multimodal sensing including high-speed imaging and acoustic sensing using hydrophones and AE sensors. The insets of (a) and (b) show the zoomed-in view of the hydrophone and AE sensor, respectively.

Fig. 4 shows the multimodal sensing during boiling testing. The signals from the thermocouples and hydrophones are connected to an NI DAQ system (chassis: cDAQ-9178, temperature module: NI 9210, sound and vibration module: NI 9230) and data are recorded using LabVIEW and exported to LabVIEW measurement (.lvm) file format. The boiling setup uses the AE sensor (MISTRAS R3a) and is connected to a data acquisition unit (1283 USB AE Node) to record the acoustic signals, including AE count, AE energy, AE hits, and AE amplitudes. Aewin software was used to process and analyze the AE raw data files. The AE sensor is attached to the outer edge of the boiling chamber and a threshold amplitude of 55 dB is used for every other test except the tests on polished MC where it is 45 dB.

The hydrophone used for recording boiling acoustics inside the liquid pool is the HTI-96 Min hydrophone series manufactured by High Tech, Inc. Table 2 provides the specifications for the particular hydrophone. These hydrophones have been used in seismic industries for ocean bottom cables and nodal systems with a working frequency range of 2 Hz to 30 kHz. The hydrophone has varying responses to the sound pressure level of 1 microPascal from minimum −240 dB to maximum −165 dB which shows high sensitivity while converting low-intensity sound signals to electric signals.

**Table 2:** Summary of Hydrophone Specifications.

Name	Additional information	Value
Sensitivity	With Pre-Amp:	Max −165 dB re: 1 V/µPa (562 V/bar) Min −240 dB re: 1 V/µPa (0.1 V/bar)
Frequency Response		2 Hz to 30 kHz
Equivalent Input Self Noise	RMS: 1 Hz – 1000 Hz	78 dB re: 1 µPa (0.08 1 µbar)
	Spectral	54 dB re: 1 µPa/√Hz @ 10 Hz 42 dB re: 1 µPa/√Hz @ 100 Hz 42 dB re: 1 µPa/√Hz @ 1000 Hz
Preamplifier Type		Voltage Mode Current Mode

Phantom VEO 710 L high-speed camera was connected to the PC via a 10GB ethernet interface for fast data transfer. Phantom Camera Control (PCC) software was used to record high-speed videos during pool boiling processes. The resolution, memory, trigger modules, frame rates, and exposure were fine-tuned for the best possible image capturing. CineViewer (CV) software was used to process the captured phantom cine video files into images which can be .JPEG, .tif, and many more. The camera was connected to NI c-DAQ 9178. The high-speed camera is synchronized with temperature and sound pressure data with BNC cables and software control used in LabVIEW which reduced the time delay to 5.69 ms [27].

MISTRAS R3a sensor manufactured by Physical Acoustics Corporation is used for sensing the acoustic waves propagated through the chamber's solid walls during boiling. Its parameters are provided in Table 3. The sensor used for the pool boiling experiments has a 30 kHz resonant response which makes it extremely useful in low frequency testing environments. The overall operating frequency range is 25–70 kHz. The sensor has been used in monitoring concretes, metal pipelines, and geologic structures. It has a wide temperature working range of −65 to 175 °C which makes it suitable for boiling experiments*.*

**Table 3:** Summary of AE sensor specifications.

Specifications	Value
Peak Sensitivity, Ref V/(m/s)	80 dB
Peak Sensitivity, Ref V/µbar	−63 dB
Operating Frequency Range	25–70 kHz
Resonant Frequency, Ref V/(m/s)	29 kHz
Resonant Frequency, Ref V/µbar	140 kHz
Temperature Range	−65 to 175 °C
Weight	41 g

### 4.3 Operation

All experimental data reported in this paper are obtained from both the steady-state and transient boiling tests. At the beginning of each experiment, water is degassed for half an hour with an opened Graham condenser. After degassing, the Graham condenser is turned off and the coiled copper tube condenser is turned on for pressure control. In the case of steady-state pool boiling tests, predefined heating power stages are reached from 0 to CHF. At each heating power, the system reaches a steady state in approximately 15 min, and raw data is collected after that. But for transient tests, a single 0 to CHF ramp-up heat is provided and data is recorded during the process. An example of raw data obtained from the pool boiling operations close to CHF initiation for two different heating conditions is plotted in the form of Fig. 1 where Fig. 1a, c, e are under steady-state heating and Fig. 1b, d, f are under transient heating.

### 4.4 Post-processing and analysis

The raw files of the AE sensor are used to extract the output in the form of a .txt file using the Physical Acoustics AE software. The hydrophone, and thermocouple data are exported as LabVIEW measurement files, the headers are removed and imported over the MATLAB code for analysis. The Heat_flux.m MATLAB code is available in the main folder for generating heat flux data based on temperature reading and contains more information commented out in it.

## Limitations

Although the study was performed trying to keep nominal background noises, the sound signal generated still was exposed to some unwanted disturbances. Some of the few limitations during the experiments are listed below:

(1) The boiling chamber has several noise-inducing operations including boiling which comprises much of the acoustics. The other noises are generated by immersion heaters, cartridge heaters, DC power supply, VARIAC (variable transformer), and so on. To mitigate the noise, a similar working environment is provided for all the experiments.
(2) One or multiple hydrophones were used at different locations to minimize position-related errors. The similar acoustic features captured from varying hydrophones show their omnidirectional properties.
(3) Tests with AE sensors put at different locations were conducted to find the most effective position which was the boiling tank bottom. Moreover, a threshold amplitude of 55 dB for polished Cu, and copper foams, and 45 dB for polished MC was employed to mitigate the probable white background noises.

## Ethics Statement

The work described above does not contain any human subjects, or animal experiments or involve data from social media platforms. All the data generated were in-house from the pool boiling facility present in the lab. This data is not published anywhere.

## CRediT authorship contribution statement

**Hari Pandey:** Conceptualization, Investigation, Methodology, Software, Formal analysis, Writing – original draft. **Changgen Li:** Writing – review & editing. **Han Hu:** Conceptualization, Supervision, Project administration, Writing – review & editing.

## Data Availability

Pool Boiling Dataset: Synchronized Acoustic, Optical, and Thermal Measurements for Steady-State and Transient Heat Loads. (Original data) (Dataverse).
